# Zoonotic Disease Programs for Enhancing Global Health Security

**DOI:** 10.3201/eid2313.170544

**Published:** 2017-12

**Authors:** Ermias D. Belay, James C. Kile, Aron J. Hall, Casey Barton-Behravesh, Michele B. Parsons, Stephanie Salyer, Henry Walke

**Affiliations:** Centers for Disease Control and Prevention, Atlanta, Georgia, USA

**Keywords:** zoonotic diseases, global health security, One Health, emerging infectious diseases, emerging health threats, global health, vector-borne infections, viruses, bacteria, zoonoses

## Abstract

Most infectious diseases that recently emerged in humans originated in animals. Besides close contact between animals and humans, other factors probably contribute to the cross-species transmission of infectious diseases. It is critical to establish effective mechanisms for coordination and collaboration between the animal, human, and environmental health sectors before new threats emerge by bringing the different sectors together to tackle endemic zoonotic diseases of greatest concern. Such multisectoral partnerships should begin by identifying priority zoonotic diseases for national engagement with equal input from the different sectors. Improvements in surveillance and data sharing for prioritized zoonotic diseases and enhancements of laboratory testing and joint outbreak response capacities in the human and animal health sectors will create and strengthen the mechanisms necessary to effectively detect and respond to emerging health threats, and thereby enhance global health security.

Zoonotic disease pathogens such as rabies virus have been causing outbreaks in humans for thousands of years (*1*). In fact, most infectious diseases in humans originate in animals, and the frequency of such transmissions has been increasing over time (*2*,*3*). Taylor et al. identified that 75% of emerging infectious organisms pathogenic to humans are zoonotic in origin (*3*). Recently emerged zoonotic diseases include globally devastating diseases such as Ebola virus disease, Middle East respiratory syndrome, highly pathogenic avian influenza, severe acute respiratory syndrome, and bovine spongiform encephalopathy (*2*–*4*). These and other zoonotic diseases affect many countries, result in high morbidity and mortality rates in humans and animals, cause disruptions of regional and global trade, and strain national and global public health resources (*5*). Newly emerging health threats are associated with substantial economic costs, including direct and indirect impacts on the healthcare system, costs associated with the actual response, and overall disruption of economic activity.

The World Bank estimated that 6 major zoonotic disease epidemics during 1997–2009 resulted in an economic loss of >$80 billion (*5*). Experiences from most recent outbreaks indicate that detecting and effectively responding to emerging epidemics require a multisectoral approach. In 2010, recognizing the need for multidisciplinary collaboration to address health threats at the human–animal–ecosystem interface, the World Health Organization (WHO), Food and Agriculture Organization (FAO), and World Organisation for Animal Health (OIE) formalized their collaboration and identified 3 priority areas of work together, 2 of which are zoonotic diseases (rabies and zoonotic influenza) (*6*). Endemic zoonotic diseases have the dual impact of causing illness and death in humans and animals as well as substantial economic loss in resource-poor societies where livestock farming is a major engine of economic growth at the household and national levels. Fortunately, proven control and prevention strategies exist for many zoonotic diseases that are most prevalent in affected communities (e.g., rabies, anthrax, brucellosis) (*7*).

To better prevent, detect, and respond to global infectious disease threats, the US government and other partners developed the Global Health Security Agenda (GHSA) with initial implementation in 17 phase 1 countries in Africa and Asia (*8*,*9*). GHSA is intended to make progress in the implementation of WHO International Health Regulations, the OIE Veterinary Services Pathway, and other similar frameworks for achieving an adequate level of preparedness to tackle emerging health threats in animals and humans. To build the necessary infrastructure and human capital, the US government and global partners allocated funds to advance GHSA across 11 action packages that included zoonotic diseases. In this paper, we describe specific steps to prevent, detect, and respond to endemic zoonotic diseases and how to leverage them to detect and effectively respond to emerging and reemerging zoonotic health threats, and thereby enhance global health security. Some of the steps have been implemented in several GHSA phase 1 countries.

## Approaches for One Health Zoonotic Disease Program Implementation

Mitigating the impact of endemic and emerging zoonotic diseases of public health importance requires multisectoral collaboration and interdisciplinary partnerships. Collaborations across sectors relevant to zoonotic diseases, particularly among human and animal (domestic and wildlife) health disciplines, are essential for quantifying the burden of zoonotic diseases, detecting and responding to endemic and emerging zoonotic pathogens, prioritizing the diseases of greatest public health concern, and effectively launching appropriate prevention, detection, and response strategies (Table). Multisectoral approaches under a One Health umbrella are more expedient and effective, and lead to efficient utilization of limited resources (*4*,*5*). 

**Table:** Implementation of zoonotic disease program activities using the One Health approach of cross-sectoral collaboration

Activity	Methods/mechanisms	Benefits
Prioritization of zoonotic disease	Semiquantitative tool	One Health multisectoral collaboration promotion and strengthening
	Workshop consisting of multisectoral teams	
		Efficient use or resources
Assessment of zoonotic disease burden	Measurement of cases of illness	Assistance in identifying priorities
	Hospitalizations	
	Disability	
	Quality-adjusted life years	
	Economic cost	
	Deaths	
Zoonotic disease surveillance	Evidence-based surveillance	Early identification of outbreaks
	Indicator-based surveillance	Opportunity for preemptive action
	Syndromic surveillance	Evaluation of prevention, detection, and response programs
	Mechanisms for data sharing and dissemination	
Joint human and animal outbreak response	Joint training of human and animal health workforce	Early detection and prompt control of zoonotic disease outbreaks
	Cross-sector emergency management systems	
	Joint risk assessments	
Development of laboratory systems in public health and veterinary sectors	Improved specimen collection, storage, and transportation	Identification of disease etiologies
	National and regional laboratory capacity development	Assistance in risk mapping of priority zoonotic diseases
	Laboratory quality and safety management	
		Surge capacity during emergencies
		Support for surveillance and outbreak response
Implementation of prevention and control strategies	Vaccination of animals and humans as needed	Protection of human and animal health
	Community and human and animal healthcare provider education	Strengthening of vaccination infrastructure
	Culling of animals (e.g., highly pathogenic avian influenza)	Education of communities to assist in emergency response

### Prioritization of Zoonotic Diseases

Developing strategies to prevent, detect, and respond to zoonotic diseases is challenging in resource-poor settings where there are other competing public health priorities. In addition, effective mitigation of their impact requires multisectoral collaborations and interdisciplinary partnerships that may take time to establish. Therefore, having all relevant sectors jointly identify zoonotic diseases of greatest concern is an essential first step for many countries. Multisectoral partnerships are easier to create if participants from multiple sectors, including human, animal (domestic and wildlife), and environmental health develop a prioritized list of zoonotic diseases to work on together and commit to sharing public- and animal-health resources. Engagement of different sectors early in the process facilitates collaboration during program implementation and ensures program ownership. In addition, systems developed to address the prioritized diseases can be leveraged to tackle other zoonotic infections and emerging health threats.

To help identify high-priority zoonotic diseases for multisectoral engagement, the One Health office at the Centers for Disease Control and Prevention (CDC) developed the One Health Zoonotic Disease Prioritization tool, a semiquantitative tool for prioritization with equal input from represented sectors, irrespective of whether reliable surveillance data are available (*10*). The tool is designed to bring together a multidisciplinary team of professionals from human, animal, and environmental health agencies and other relevant sectors with a common goal of developing country-specific criteria for ranking zoonotic diseases of greatest national concern. The tool has been used to select zoonotic diseases for further programmatic activity in multiple countries in the implementation of the zoonotic disease action package of GHSA (*11*,*12*). Typically, the prioritization is performed by trained facilitators during a workshop with voting members from multiple ministries covering human, animal, and environmental health and from multinational organizations (e.g, FAO, WHO, OIE), academic institutions, and other partners working in the area of zoonotic diseases (e.g., CDC, US Agency for International Development). The country’s government ministries should select participants. In countries that have conducted prioritization workshops, CDC provided training to in-country workshop facilitators to promote country ownership of the process. Minimizing the role of external facilitators helps to retain objectivity in the process and allow decision making by the host country representatives.

### Assessing Burden of Zoonotic Diseases

Accurately estimating the burden of zoonotic diseases is a critical step in both identifying public- and animal-health priorities and assessing the impact of prevention and control strategies, including potential economic effects on the food supply, such as with avian and swine influenza viruses. Metrics for human zoonotic disease burden may include numbers of cases of illness, hospitalizations, deaths, disability, or quality adjusted life years, and economic impacts such as healthcare-associated costs and lost productivity. Some of these metrics can also be used to assess animal health burden. In countries where zoonotic disease data may not be readily available, the burden of different zoonotic diseases could be better ascertained by conducting studies in selected regions. Such studies may focus on zoonotic diseases selected in the prioritization process or diseases that are deemed more prevalent on the basis of limited epidemiologic or clinical data. Estimation of disease burden should involve studies in humans and affected or implicated animal species. Conducting ecologic and wildlife studies may be necessary to define risk to humans from selected zoonotic pathogens in animal reservoirs or arthropod vectors. Investigators should consider using existing databases or laboratory specimens, such as banked sera collected as part of HIV indicator surveys, to quantify the potential risks to humans of some zoonotic diseases.

### Zoonotic Disease Surveillance in Animals and Humans

A rapid and effective response to endemic and emerging zoonotic diseases relies heavily on a timely and efficient surveillance and reporting system (*13*). Surveillance in animals and humans is critical for early identification and possible prediction of future outbreaks, allowing for preemptive action. Components of effective surveillance include establishing event-based and indicator-based surveillance, and adequate laboratory capacity in both public health and animal health laboratory systems. Training epidemiologists and establishment of effective laboratory systems are critical for a successful zoonotic disease surveillance program.

An effective surveillance system may require the following: standard case definitions for priority zoonotic diseases under surveillance, based on existing guidance from global human and animal health organizations such as WHO, CDC, OIE, and FAO; evaluation of existing national surveillance systems to determine their timeliness, effectiveness, and usefulness; new or refined surveillance and reporting systems and linkages to share data between public health and animal health agencies and other relevant sectors (*14*); evaluation of potential electronic disease reporting mechanisms, including the use of smartphone technologies; establishment of surveillance data dissemination platforms (which may include regular reports and publications) to provide awareness and feedback to human and animal health agencies and other stakeholders; evaluation of available diagnostic tests and appropriate testing capabilities in central and regional public health and animal health laboratories; and establishment of a national emergency management system, such as an Emergency Operations Center, to assist in coordinated zoonotic disease surveillance, response to zoonotic disease outbreaks, and prevention and control efforts across relevant sectors.

### Laboratory Systems

Timely, accurate, and reliable laboratory tests are critical for building outbreak response capacities, identify etiologies of disease, and to monitor endemic and emerging zoonotic diseases in humans, domestic and food animals, and wildlife. Well-functioning and separate national public health and animal health laboratory systems are essential to identify etiologic agents so that appropriate prevention, detection, and response strategies can be implemented. Laboratories should be an integral part of the public health infrastructure with a system for rapid testing of prioritized samples and timely sharing of results. Successful and sustainable laboratory systems require strategic interagency planning across sectors and building on existing capacities in country to standardize laboratory methods, prioritize laboratory resources, and develop information sharing channels (*15*). A requirement for ensuring testing quality is commitment from the top levels of management to provide the necessary resources to sustain the functional roles of the laboratory in an environment that supports quality and safety. The roles and responsibilities of all human and animal laboratory staff need to be defined, documented, and communicated, and written policies and procedures should be available and understood. In addition, all laboratory staff should be trained on these policies and procedures to ensure they are executed in a consistent and reliable manner. Accurate and reliable test results depend on having a sample that has been collected, stored, and transported correctly; sample requirements vary by the disease and suspected pathogen. Laboratories should be designed to optimize workflow, support the quality of testing, and protect the safety of laboratory staff and the community. Regularly conducted proficiency testing helps to monitor the quality and performance of the laboratory.

Critical human and animal laboratory systems that countries need to establish or expand include central and regional laboratory capacity; specimen referral systems for rapid, safe, and reliable specimen transport; laboratory training programs that promote workforce development and retention; and affordable, flexible laboratory accreditation schemes to ensure lab quality (*16*). Opportunities for mentored relationships with reference laboratories or private partnerships should be encouraged (*16*). Laboratories may assist in determining disease burden and characterization of human, animal, and ecologic drivers of disease spillover from animals to humans to optimize models for predicting disease emergence (e.g., risk mapping).

### Outbreak Response Using One Health Approach

A successful zoonotic disease outbreak response requires 1) the ability to detect the outbreak using established surveillance systems including event-based reporting; 2) adequate laboratory capability to confirm the outbreak etiology; 3) a workforce trained to respond and perform descriptive and analytical epidemiology for animal and human diseases; 4) the ability to implement appropriate control and prevention measures; and 5) an outbreak and emergency management system in place to coordinate multisectoral response activities at the national to subnational levels. Involvement of all relevant stakeholders is crucial, including those in human, animal, and environmental health sectors. Outbreak response activities are best supported by an overarching operations framework that clearly identifies the roles and responsibilities of key institutions and officials for all relevant sectors and provides direction for coordination of activities at the local and national levels. Countries should establish functional cross-sector coordination and communication pathways before an outbreak occurs. Multisectoral collaboration is easier during an emergency if agencies had already been collaborating in joint priority setting and actively working together to address prioritized zoonotic diseases.

Early detection of an impending human outbreak may in some instances be achieved through detection of an increase in disease in animal populations, such as livestock and wildlife populations. Detection of an outbreak or an increase in case count of a zoonotic disease by the wildlife, livestock, or public health agency should trigger enhanced surveillance by the other agencies. This detection can only occur if there is effective communication between the different sectors. Outbreak response protocols or national strategies should be developed for priority zoonotic diseases that specifically address coordination of activities, data sharing (including how to integrate animal, human, and environmental health information), trigger points or threshold for action, and roles and responsibilities of each stakeholder. Establishing joint training opportunities for animal and human health workers will facilitate information sharing and enhance collaboration for effective prevention, detection, and response programs. When possible, joint simulation exercises can be conducted to demonstrate proficiency of a response and adequate interagency and multisectoral collaboration.

### Prevention and Control of Zoonotic Diseases

The prevention and control strategies of zoonotic diseases will vary by disease and availability of proven interventions (Figure). Some of the zoonotic diseases most prevalent in resource-limited areas are vaccine preventable (e.g., rabies, brucellosis, anthrax). Therefore, implementation of routine immunization programs may be needed for disease prevention. Depending on the disease, this may be primarily human vaccination or vaccination of livestock or other domestic animals. For some diseases, such as highly pathogenic avian influenza, prevention and control may involve large-scale culling and effective biosecurity programs. For diseases such as anthrax and rabies, preemptive vaccination of animals will prevent outbreaks in the animal population while at the same time protecting humans. In others (e.g., Rift Valley fever), disease outbreaks in animals may be the first signal to start implementation of prevention programs such as ring vaccination of animals. Waiting until an outbreak is detected in humans can be costly to the lives of animals and humans and can strain limited public health resources.

**Figure:**
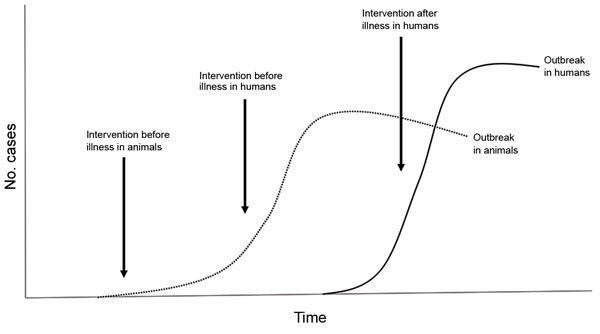
Opportunities for intervention to prevent and control endemic and emerging zoonotic diseases.

Effective human and animal disease surveillance systems are critical for early detection and response, for planning prevention and control programs, and to evaluate the effectiveness of control and prevention strategies. Timely and effective communication and collaboration between human and animal health agencies are essential to develop disease prevention and control strategies involving both human and animal populations. As part of an effective response, countries should consider developing and evaluating communication strategies to educate human and animal healthcare providers and the general population on zoonotic disease transmission and prevention. Community education programs may include safe farming and biosecurity measures, animal slaughtering practices, understanding animal contact and exposure risks, and use of personal prevention measures to avoid or reduce exposure to vectorborne and other zoonotic diseases. Livestock and poultry are key sources of food and livelihood, and important economically for trade; prevention strategies that target zoonotic diseases associated with food animals must be compatible with the needs of the communities that are economically dependent on those animals.

Communicating effectively regarding prevention strategies will also enhance engagement in future outbreak control efforts because the communities will better understand the reasons behind any intervention. Similarly, a well-informed population can serve as an early alert system, notifying appropriate authorities about possible cases of disease in humans or animals. For zoonotic diseases with potential domestic and food animal reservoirs, important strategies in disease control can include animal vaccination, vector control, test and treat, or cull programs, and effective biosecurity measures. The development and implementation of cost-effectiveness and cost-benefit models to evaluate and refine disease prevention and control methods and programs will ensure effective use of resources; evaluations may include the negative effects culling has on societal well-being and livelihood of farmers.

## Conclusions

Effective zoonotic disease prevention, detection, and response requires close collaboration, including well-defined roles and responsibilities among the animal, human, and environment health sectors. Such collaborations can help reduce illness and deaths in animals and humans and minimize their social and economic impact at the household and national levels. In most countries, animal health and human health decision makers are located within different ministries. Establishing multisectoral One Health partnerships across agencies and with interdisciplinary personnel at the national, subnational, and local levels (including government departments responsible for health, agriculture, veterinary services, environment, and laboratories) can strengthen zoonotic disease detection and response activities. These structures must be in place before an outbreak, epidemic, or pandemic occurs to have an effective, coordinated public- and animal-health response. Countries that lack a well-functioning coordination mechanism could fail to rapidly detect and effectively respond to emerging health threats, which could spread to other countries and threaten global health security. 

Countries should consider convening regular cross-sectoral meetings to build multisectoral and interdisciplinary relationships, encourage transparency, and combine efforts across agencies. Developing mutually agreed-upon standard operating procedures is essential. Identifying designated points of contact ensures improved coordination across sectors, allowing for quicker collaborative response to zoonotic disease outbreaks. Additional benefits of establishing a formal, multisectoral coordination mechanism include identifying high-priority research areas and developing training opportunities for interdisciplinary outbreak response teams. Multisectoral collaborations should also be established at subnational levels. Identifying One Health focal points at the local, district, and regional levels is critical and the list of these designated contacts should be shared among sectors. These approaches will enhance cross-sectoral utilization of limited resources while leveraging each sector’s capabilities for improved prevention, detection, and response of zoonotic diseases. 

In some countries, formal, national collaborative One Health coordinating mechanisms were established to facilitate multisectoral engagement. Examples include the Zoonotic Disease Unit in Kenya, the Zoonotic Disease Secretariat in Cameroon, and the Guidelines for Coordinated Prevention and Control of Zoonotic Diseases in Vietnam (*17*). Creation of such mechanisms with dedicated financial and human resources will facilitate outbreak detection and response, prevention and control of high-priority endemic zoonotic diseases, and early detection and response to emerging health threats. They also allow countries to develop shared visions to maximize impact and build in measurements for success, and help design an overall plan for sustainability of cross-sectoral collaborations.
